# A PD-1 Inhibitor Induces Complete Response of Advanced Bladder Urothelial Carcinoma: A Case Report

**DOI:** 10.3389/fonc.2021.671416

**Published:** 2021-06-18

**Authors:** Jianzheng Wang, Qingli Li, Huifang Lv, Caiyun Nie, Beibei Chen, Weifeng Xu, Tiejun Yang, Yinping Zhang, Shuiping Tu, Xiaobing Chen

**Affiliations:** ^1^ Department of Medical Oncology, Affiliated Cancer Hospital of Zhengzhou University, Henan Cancer Hospital, Zhengzhou, China; ^2^ Department of Oncology, Renji Hospital, School of Medicine, Shanghai Jiaotong University, Shanghai, China; ^3^ Department of Urology Surgery, Affiliated Cancer Hospital of Zhengzhou University, Henan Cancer Hospital, Zhengzhou, China; ^4^ Department of Pathology, Affiliated Cancer Hospital of Zhengzhou University, Henan Cancer Hospital, Zhengzhou, China

**Keywords:** PD-1, complete response, advanced, urothelial carcinoma, case report

## Abstract

The prognosis of patients with advanced urothelial carcinoma is dismal. Platinum-based chemotherapy is still the main first-line treatment for advanced urothelial carcinoma, while immunotherapy can be used as a first-line treatment option for people who cannot tolerate platinum. Immunotherapy is preferred in the second-line treatment of bladder urothelial carcinoma. PD-1 inhibitors (Pembrolizumab, nivolumab and atezolizumab) and PD-L1 inhibitors (Ddurvalumab and avelumab) have not been approved for the treatment of advanced urothelial cancer in China. We describe a patient with advanced urothelial carcinoma experienced disease progression after gemcitabine chemotherapy. Following a treatment of domestic PD-1 inhibitor (sintilimab), the patient achieved a durable complete response with mild toxicity. This case indicates that PD-1 inhibitor sintilimab might be a second-line treatment choice for advanced urothelial carcinoma.

## Background

Bladder urothelial carcinoma is the most common malignant tumor of the urinary system in China. Advanced urothelial carcinoma is sensitive to platinum-based chemotherapy, with an effective rate of up to 50% (1, 2). However, some patients cannot tolerate cisplatin-based chemotherapy. Therefore, the treatment of advanced urothelial carcinoma is divided into non-cisplatin chemotherapy and cisplatin chemotherapy according to platinum tolerance. In general, most of the non-cisplatin chemotherapy is not satisfied. Therefore, for those who cannot tolerate cisplatin therapy, cisplatin-free chemotherapy or other treatments are recommended. Gemcitabine, as one of the chemotherapeutic drugs for advanced urothelial carcinoma, is used alone for the first-line treatment of bladder urothelial carcinoma. The results of a phase 2 study showed that the objective effective rate was 24%-44%, of which the complete remission rate was 8%-17%, and the median overall survival time was 8-13.5 months (3). For patients with advanced bladder urothelial carcinoma who cannot tolerate platinum-based chemotherapy, immunotherapy is also recommended.

Immune checkpoint inhibitors represented by PD-1/PD-L1inhibitors significantly improve the objective and effective rate of the second-line treatment of advanced urothelial carcinoma compared with traditional chemotherapy, and opened a new chapter in the second-line treatment of advanced bladder cancers. In particular, the randomized controlled phase 3 clinical study of pembrolizumab and chemotherapy (KEYNOTE045) showed that immunotherapy improved the overall survival, and established the status of immunotherapy in the second-line treatment of advanced urothelial carcinoma (4). Sintilimab is a monoclonal antibody against programmed cell death protein 1. It is developed by Innovent Biologics and Eli Lilly and Company and has been approved to treat relapsed or refractory classical Hodgkin lymphoma in patients who have undergone two or more lines of systemic chemotherapy by the National Medical Products Administration of China. It has been reported in number of literature and shows satisfying anti-tumor effect (5).

## Case Presentation

**Figure 1:**
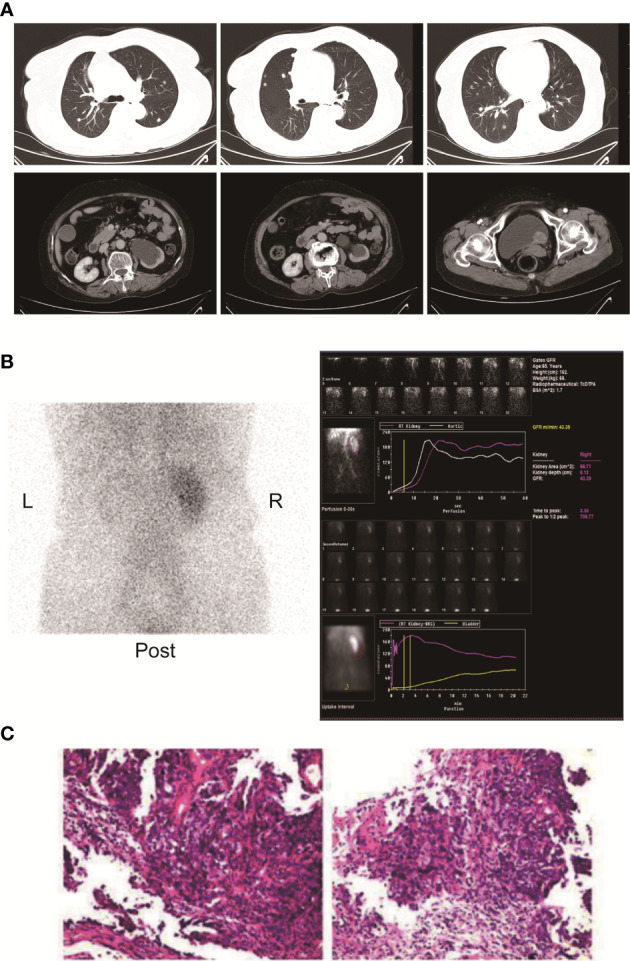
**(A)** Enhanced CT of the chest and pelvis showed multiple round nodules in both lungs with clear boundaries. A dense mass of soft tissue is seen at the entrance of the ureter on the left side of the bladder. The size is about 25mm*22mm. **(B)** Renal dynamic imaging and glomerular filtration. 1. Renal blood perfusion image: After intravenous "projectile" injection of imaging agent 99m-Tc-DTPA, renal blood perfusion imaging was performed. After imaging of the abdominal aorta, the right kidney was immediately imaged, and the position, shape and the size is roughly normal, and the radioactivity distribution is roughly even; the left kidney is not seen. 2. Renal function shows: After renal blood flow perfusion, the left kidney is not visible; the shadow of the right kidney gradually increases, and the imaging of right kidney reaches a peak at 3.35 minutes. After that, the shadow of the renal cortex diminishes, the shadow of the renal medulla increases, and the renal excretion process. There was no abnormal reflex concentration in the right renal pelvis; after 5 hours of delay, the right kidney was lightened, there was no radioactive concentration in the right renal pelvis and ureter, and the left kidney was slightly developed. 3. Renal functional curve shows: the curve of the right kidney is roughly normal; the peak time of the right kidney is 3.35min; the GFR of the right kidney is: 43.39ml/min. **(C)** Pathological results of HE staining on cystoscopy biopsy.

**Figure 2:**
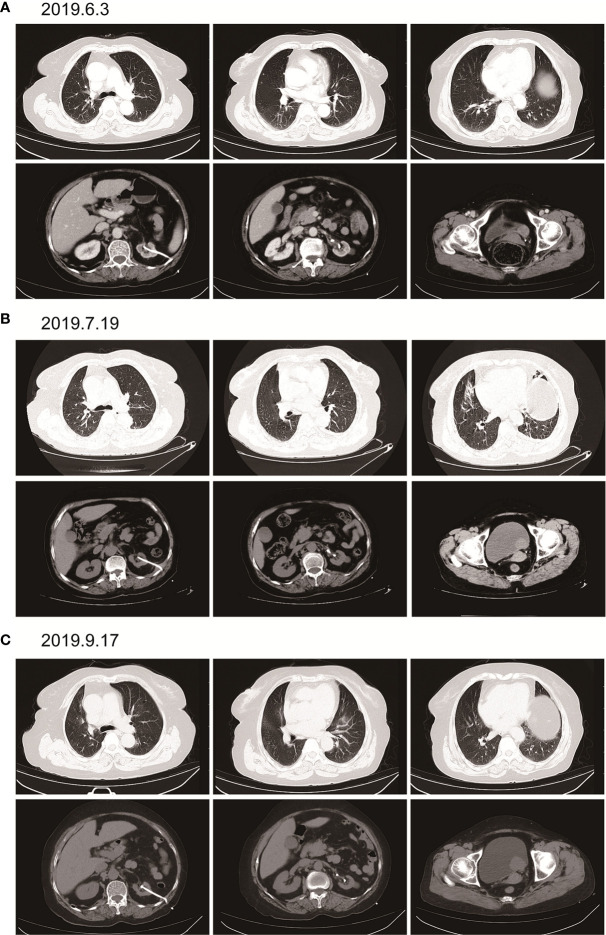
Imaging examination after single-agent gemcitabine chemotherapy. **(A)** (2019.6.3) after two cycles of gemcitabine. Multiple round small nodules are seen in both lungs, with clear borders and reduced size compared to the previous ones. A dense mass of soft tissue at the entrance of the ureter on the left side of the bladder, with a size of about 25mm*22mm, and a clear boundary. **(B)** (2019.7.19) after 4 cycles of gemcitabine. Multiple round-shaped nodules in both lungs, with clear boundaries and slightly smaller than before. A dense mass of soft tissue at the entrance of the ureter on the left side of the bladder, with a size of about 25mm*22mm, with a clear boundary. **(C)** (2019.9.17) after 6 cycles of gemcitabine. Multiple round nodules in both lungs, with clear boundaries, and some were slightly larger than before. The soft tissue density mass at the entrance of the ureter on the left side of the bladder is shadowed, about 37mm*30mm in size, with a clear boundary and larger than before.

Here, we present a case of advanced bladder urothelial carcinoma. A 66-year-old female patient was diagnosed with bladder occupying mass, left hydronephrosis, and left ureter dilatation in a local hospital due to painless hematuria for 2 months. The patient was then hospitalized in Henan Cancer Hospital. The results of enhanced CT (2019.4.4) showed: 1. Thickening of the lower ureter and nodules at the entrance of the vesicoureter, about 25mm*22mm in size, with fluid in the dilated upper ureter and renal pelvis, and possible atypical cell carcinoma. 2. There are multiple lymph nodes in the retroperitoneum and between the liver and stomach. 3. Multiple metastases in both lungs. 4. There are multiple swollen lymph nodes in the running area of the left iliac vessels (**Figure 1A**). Renal dynamic imaging and glomerular filtration rate were measured with 99m-Tc-DTPA. Diagnostic considerations: 1. No obvious abnormality in blood perfusion of the right kidney. 2. The right glomerular filtration function is normal. 3. Poor excretion in the upper right urinary tract. 4. No function in the left kidney (**Figure 1B**). In order to relieve the symptoms of left kidney compression, a percutaneous external renal pelvic drainage tube was placed on April 11, 2019. Cystoscopy was performed, and the pathological results of biopsy showed urothelial carcinoma. There was no clear muscle infiltration in the submitted tissues(**Figure 1C**). Immunohistochemistry: P63+,CK20+,CK7+, CyclinD1+, Ki-67: 80% (**Supplementary Figure 1**). The final diagnosis was stage IV bladder urothelial carcinoma with multiple lung metastases. Because the patient has only one normal kidney left. According to the patient’s age, physical fitness score and considering the nephrotoxicity of cisplatin, we choose gemcitabine (1000mg/m^2^, d1、8, q21d) as a single agent for the first-line treatment. After two cycles of gemcitabine treatment, the efficacy of chemotherapy was evaluated as SD (stable disease) (**Figure 2A**). The efficacy of chemotherapy remained as SD after four cycles of treatment (**Figure 2B**) and it became PD (progression disease) after six cycles of treatment (**Figure 2C**).

**Figure 3:**
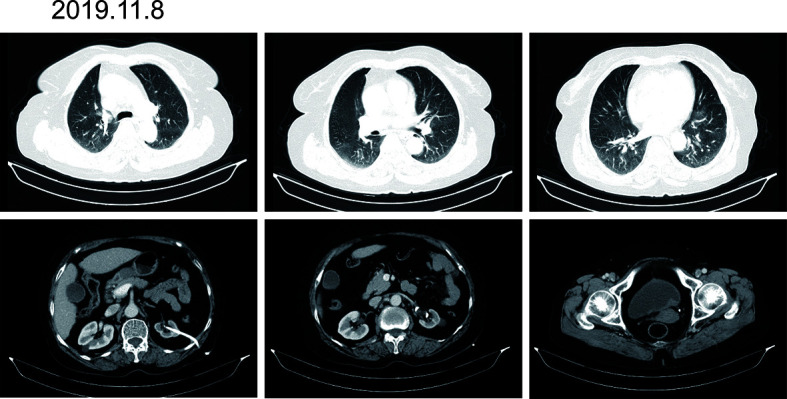
Imaging after three cycles of Sintilimab immunotherapy. There are multiple round-shaped nodules in both lungs, with clear boundaries, and some are smaller than before. The local soft tissue shadow at the entrance of the ureter on the left side of the bladder thickens, forming soft tissue nodules, the size is about 24mm*10mm, which is smaller than before.

**Figure 4:**
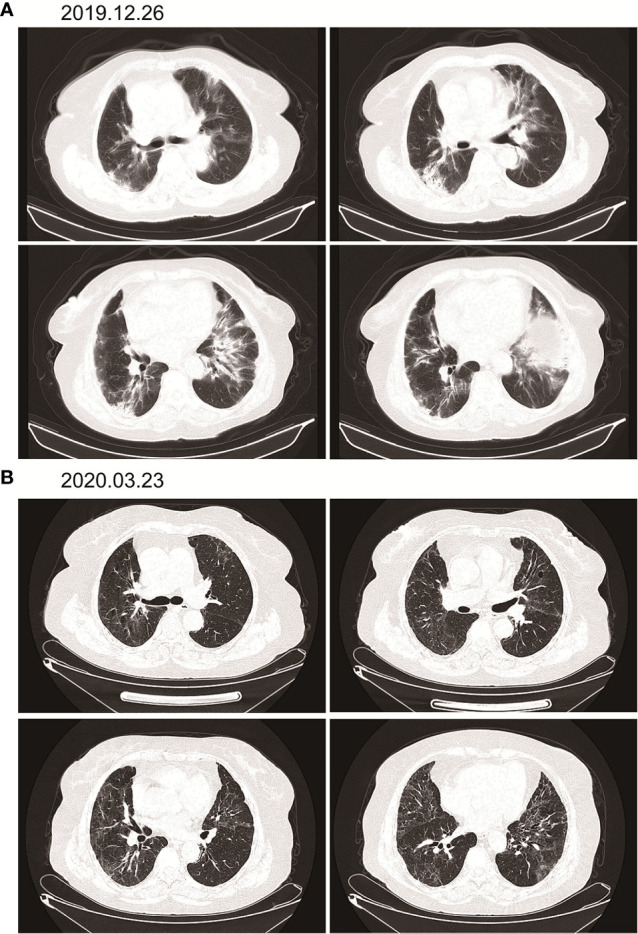
Immunotherapy-related pneumonia before and after methylprednisolone therapy. **(A)** There are cords and patchy shadows in both lungs, with blurred boundaries and inflammatory changes, that are bigger than before. **(B)** Frequent strand shadows and patchy shadows are seen in both lungs, and the boundary is blurred, and the area is smaller than before.

**Figure 5:**
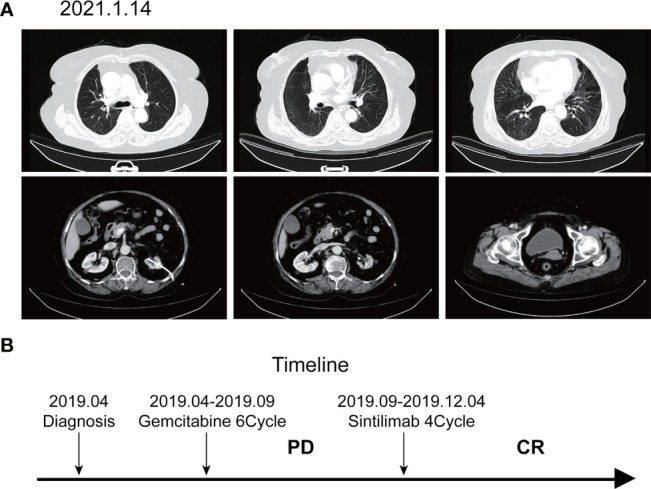
Imaging after discontinuation of Sintilimab for eight months. **(A)** The inflammation in both lungs disappeared, the metastases in both lungs disappeared, and the soft tissue shadow at the entrance of the ureter on the left side of the bladder disappeared. **(B)** Timeline of disease status and corresponding treatment regimens.

According to the patient’s age, economic status, PD-1 donation policy and family’s wishes, Sintilimab (200mg, on day1, q21d) was used for the second-line treatment for three cycles on 2019.9.20、2019.10.16 and 2019.11.12. The treatment efficacy is evaluated as PR (Partial response) (**Figure 3**). Then Sintilimab was given for the fourth cycle of immunotherapy on 2019.12.04. One week later, on December 10, 2019, the patient developed chest tightness and shortness of breath, accompanied by gradual increase in dyspnea. The local hospital gave oxygen inhalation and anti-asthmatic treatment, but the symptoms did not alleviate. Chest CT examination considered infectious lesions of both lungs. Serological examination ruled out tuberculosis or Aspergillus infections. Subsequently, the patient was transferred to our hospital for further treatment. On admission, her blood oxygen saturation was 75%, and his skin was cyanotic. The breath sound of auscultation in both lungs is thick, and the wheezing sound can be heard. The results of chest CT examination on 2019.12.26 showed pneumonia in both lungs, that are more severe than earlier images (**Figure 4**). The patient was suspected to had developed grade 3 immunotherapy-related pneumonia, and was given an intravenous infusion of methylprednisolone at 1 mg/(Kg.d) for 72 hours. After 3 days, the patient’s chest tightness symptoms improved, and oxygen saturation reached 94%. The methylprednisolone was changed to oral administration and the dose was gradually reduced by 5-10mg per week. By March 23, 2020, methylprednisolone was reduced to 10mg/d orally, at which time the blood oxygen saturation had reached 96%. We performed chest CT to observe the changes of pneumonia, and the results showed that pneumonia of both lungs had improved significantly (**Figure 4B**). On January 14, 2021, the patient underwent whole-body enhanced CT evaluation. To our surprised, the results showed that the patient’s lung metastases disappeared, and the local soft tissue shadow at the entrance of the ureter on the left side of the bladder disappeared completely (**Figure 5A**). The patient achieved progression-free survival for more than one year (**Figure 5B**). The current condition is generally good.

## Discussion

Bladder cancer refers to a malignant tumor occurred on the bladder mucosa. It is the most common malignant tumor of the urinary system and one of the ten most common tumors in the body. Bladder cancer is the number one urogenital tumors in China (6). Cisplatin-based chemotherapy is the standard therapy for stage IV bladder malignancy, which can increase the overall survival but rarely results in complete remission. The most significant breakthrough in cancer therapy over the last decade was the development of immunotherapy (7). Immunotherapy is approved as a second-line treatment for metastatic urothelial carcinoma. Its use as a first-line agent is limited to patients who are ineligible for cisplatin-based treatments. Five drugs are approved by the Food and Drug Administration of USA for metastatic urothelial carcinoma including three Programmed cell-death protein 1 (PD-1) inhibitors and two programmed cell-death ligand 1 (PD-L1) inhibitors for patients whose tumor progressed during or after platinum-based therapy. However, only two drugs were approved based on phase III clinical trials—pembrolizumab and atezolizumab, of which only KEYNOTE study performed with pembrolizumab showed overall survival benefits.

Atezolizumab and pembrolizumab are the Food and Drug Administration–approved checkpoint inhibitors in cisplatin-ineligible patients. However, the PD-1 antibody approved in China to treat classical Hodgkin’s lymphoma is sintilimab, which is still under clinical trials to test its efficacy and safety in solid tumors (8, 9). There is no report on the clinical effect of this immune checkpoint inhibitor (ICI) sintilimab in advanced bladder cancer. Considering the age of the patient and the wishes of his family members, it is recommended that immunotherapy be performed as soon as possible. As a representative domestic PD-1 monoclonal antibody, sintilimab has a 10 times and 50 times higher affinity for human PD-1 receptors than pembrolizumab and nivolumab, respectively (10).

Immune checkpoint blockade can cause inflammatory reaction by enhancing the activity of the immune system, and these side effects are often referred to as immune-related adverse events (11). Immune-related pneumonia is a rare but fatal serious adverse event. Data from clinical studies show that the incidence of pneumonia in patients treated with PD-1/PD-L1 inhibitors is less than 5%, and the incidence of pneumonia above grade 3 is up to 1.5% (12–16). PD-1 inhibitors and PD-L1 inhibitors caused 3.6% and 1.3% of all grades of pneumonia, respectively, and the incidence of severe pneumonia was 1.1% and 0.4% (15, 17). So far, there is no convincing evidence that PD-1 inhibitors and PD-L1 inhibitors have a significant difference in the incidence of adverse events in the respiratory system. Immune-related pneumonia may occur at any time, but compared with other irAEs, pneumonia occurs relatively late, with a median occurrence time of 2.8 months. This patient developed immunotherapy-related pneumonia 90 days later after treatment. In general, the onset of pneumonia in patients with combination therapy occurs earlier. Also, the onset of pneumonia in patients with NSCLC occurs later than that of malignant melanoma (18). The high-risk population of immune-related pneumonia include: 1. NSCLC patients with positive driver gene sensitive mutations treated with EGFR-TKI combined with immune checkpoint inhibitors (19, 20); and 2. Previously diagnosed with chronic obstructive pulmonary disease (COPD), pulmonary fibrosis or patients with active lung infections (13, 20, 21). Gemcitabine can damage the lungs and cause lung diseases such as interstitial pneumonia (22). This patient was treated with gemcitabine in first-line chemotherapy. Whether there is a connection between gemcitabine and immunotherapy-related pneumonia caused by subsequent use of immune checkpoint inhibitor is worthy of further exploration.

At present, the mechanism of immune-related adverse reactions has not been fully understood. It is generally thought to be the inflammatory response of the immune system to specific organs and tissues. The adverse reactions related to immunotherapy may have a certain correlation with the treatment efficacy, but they are not positively correlated. Immune-related adverse reactions, especially rash or itching, may implicate better efficacy. The predictor of efficacy of immunotherapy has always been a hot research topic. Some previous retrospective analyses have suggested that adverse reactions related to immunotherapy can predict the efficacy of treatment. Early appearance of rash and itching is a strong predictor of the efficacy of nivolumab (23). Whether immunotherapy-associated pneumonia is positively correlated with the curative effect is currently inconclusive. However, there are some case reports that the appearance of immunotherapy-related pneumonia suggests better efficacy, even complete remission, and long-term survival of patients (24, 25). The mechanism by which irAEs can predict the efficacy of PD-1/PD-L1 needs to be further clarified in the future.

In performing immunotherapy, attentions must not only be paid to its efficacy, but also its side effects. There are evidence show that some patients might not benefit from immunotherapy, instead, the tumor grows bigger and their survival time might shortened after receiving immunotherapy. This phenomenon is called hyperprogressive disease (HPD) (26, 27). MDM2/4 gene and DNMT3A gene mutations are closely related to HPD. However, these HPD indicators are very immature and need a lot of clinical data to support (28).

In conclusion, complete regression of advanced bladder cancer following immunotherapy is an extremely rare but spectacular event. The precise mechanism of this phenomenon remains a mystery, and no specific factor seems to be responsible for tumor regression. Additional research is warranted to explain the possible mechanisms. Knowledge of these mechanisms may help elucidate the nature of bladder cancer and the disease management.

## Data Availability Statement

The original contributions presented in the study are included in the article/**Supplementary Material**. Further inquiries can be directed to the corresponding authors.

## Author Contributions

JW, XC, and TY treated the patient. JW, HL, CN, BC, and WX collected the data. JW, QL, YZ, and ST analyzed the data and wrote the original draft. All authors contributed to the article and approved the submitted version.

## Funding

This work was financially supported by the Science and Technique Foundation of Henan Province (No. 202102310121 for JW), 1000 Talents Program of Central plains (No. 204200510023 for XC), State Key Laboratory of Esophageal Cancer Prevention & Treatment (No. Z2020000X for XC).

## Conflict of Interest

The authors declare that the research was conducted in the absence of any commercial or financial relationships that could be construed as a potential conflict of interest.
