# Supplementary material for: A PD-1 Inhibitor Induces Complete Response of Advanced Bladder Urothelial Carcinoma: A Case Report
Source: Front Oncol. 2021 Jun 18;11:671416. doi: 10.3389/fonc.2021.671416 (PMC8249845; doi:10.3389/fonc.2021.671416)
Supplement: Supplementary file 1 [file DataSheet_1.docx]

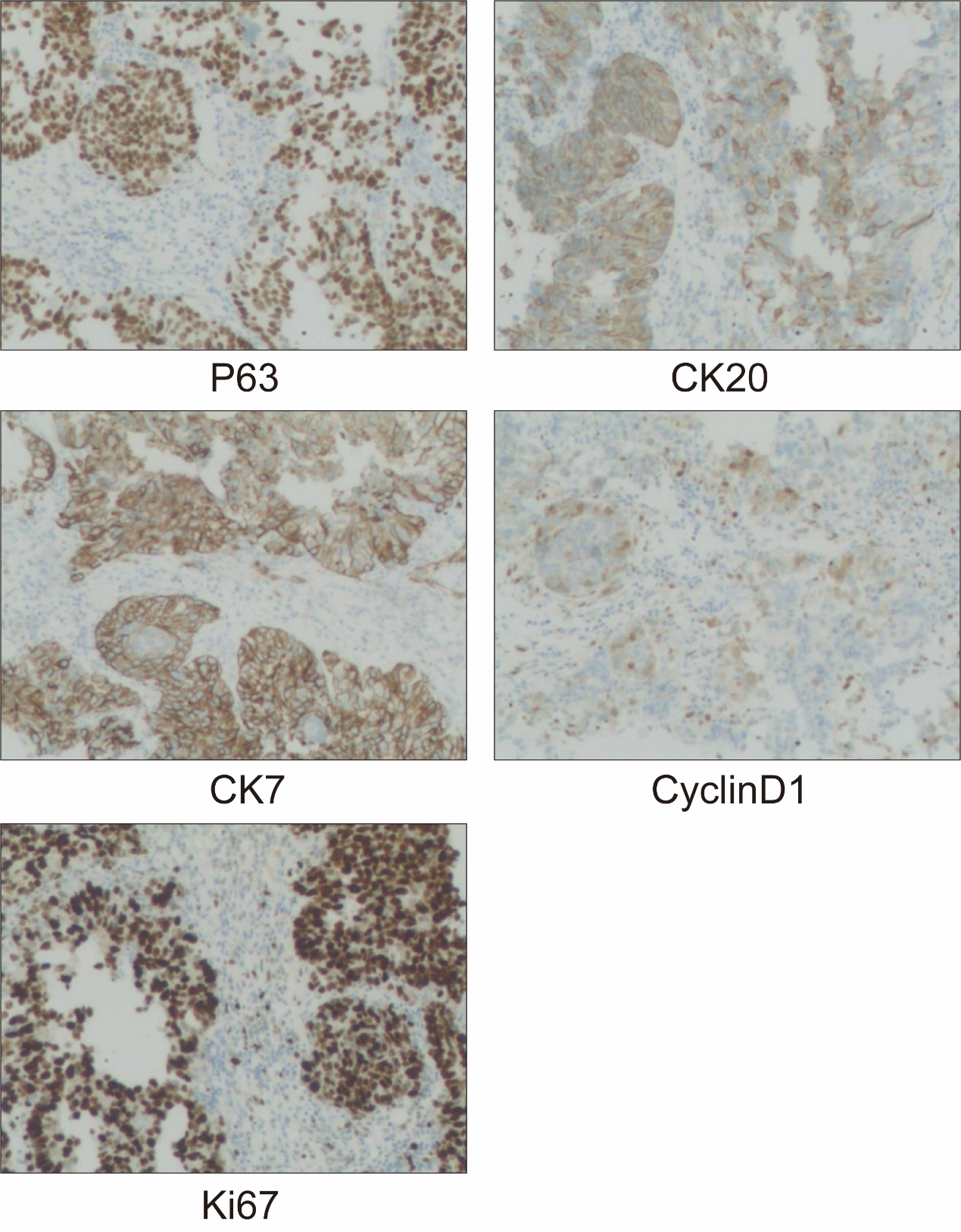


**Supplementary Figure1.** **Immunohistochemical indexes of urothelial carcinoma.** **P63+, CK20+,CK7+, CyclinD1+, Ki-67: 80%.**
